# The Common Task

**Published:** 1908-02-08

**Authors:** 


### THE COMMON TASK.

The Store Rooms.

We have often wondered over the neglected appearance of the storerooms in institutions otherwise apparently well managed. Goods of the most various description will be huddled together—in one corner piles of articles packed so close that it would take half an hour to extricate anything which was wanted; on another side empty shelves containing a few brooms; bales of goods which have not been sorted or even unpacked; a general air of hurry and neglect. It can hardly be supposed that this indifference to the arrangement of stores can exist side by side with true economy. The good housekeeper cares intensely how her stores are disposed. It gives her real satisfaction to see fresh things arrive, and she has a destined place for each, in which she is satisfied that they are well placed. She likes to get a good view of all she possesses. She has a table conveniently situated for conducting all sorts of little matters connected with the business of keeping shop. She has a receiving place, even if the storerooms are but a converted cellar, and she has a counter for issuing stores. Her appliances number the indispensable scales, books for entries and counterfoils for issuing what is wanted, scissors, string, paste-pot, blue pencil for marking pots, and a little red tape, both real and symbolical. The hours she spends in the storerooms are some of the pleasantest in her day, for she is fairly uninterrupted, and she feels the strands of the economist tighten in her grasp. The sister who finds in the storeroom no charm may be an excellent nurse; she will never make a good housekeeper.

